# Mutant SPART causes defects in mitochondrial protein import and bioenergetics reversed by Coenzyme Q

**DOI:** 10.1098/rsob.230040

**Published:** 2023-07-12

**Authors:** Chiara Diquigiovanni, Nicola Rizzardi, Antje Kampmeier, Irene Liparulo, Francesca Bianco, Bianca De Nicolo, Erica Cataldi-Stagetti, Elisabetta Cuna, Giulia Severi, Marco Seri, Miriam Bertrand, Tobias B. Haack, Adela Della Marina, Frederik Braun, Romana Fato, Alma Kuechler, Christian Bergamini, Elena Bonora

**Affiliations:** ^1^ Department of Medical and Surgical Sciences, University of Bologna, Bologna 40138, Italy; ^2^ Center for Applied Biomedical Research (CRBA), University of Bologna, Bologna 40138, Italy; ^3^ IRCCS Azienda Ospedaliero-Universitaria di Bologna, Bologna 40138, Italy; ^4^ Department of Pharmacy and Biotechnology, University of Bologna, Bologna 40126, Italy; ^5^ Institut für Humangenetik, Universitätsklinikum Essen, Universität Duisburg-Essen, Essen 45122, Germany; ^6^ Department of Veterinary Sciences, University of Bologna, Bologna 40064, Italy; ^7^ Institute of Medical Genetics and Applied Genomics, University of Tübingen, Tübingen 72076, Germany; ^8^ Center for Rare Diseases, University of Tübingen, Tübingen 72076, Germany; ^9^ Department of Pediatric Neurology, Centre for Neuromuscular Disorders, Centre for Translational Neuro- and Behavioral Sciences, University Duisburg-Essen, Essen 45122, Germany

**Keywords:** SPG20, Spartin, bioenergetics, mitochondrial protein import, Coenzyme Q

## Abstract

Pathogenic variants in *SPART* cause Troyer syndrome, characterized by lower extremity spasticity and weakness, short stature and cognitive impairment, and a severe mitochondrial impairment. Herein, we report the identification of a role of Spartin in nuclear-encoded mitochondrial proteins. *SPART* biallelic missense variants were detected in a 5-year-old boy with short stature, developmental delay and muscle weakness with impaired walking distance. Patient-derived fibroblasts showed an altered mitochondrial network, decreased mitochondrial respiration, increased mitochondrial reactive oxygen species and altered Ca^2+^ versus control cells. We investigated the mitochondrial import of nuclear-encoded proteins in these fibroblasts and in another cell model carrying a *SPART* loss-of-function mutation. In both cell models the mitochondrial import was impaired, leading to a significant decrease in different proteins, including two key enzymes involved in CoQ10 (CoQ) synthesis, COQ7 and COQ9, with a severe reduction in CoQ content, versus control cells. CoQ supplementation restored cellular ATP levels to the same extent shown by the re-expression of wild-type SPART, suggesting CoQ treatment as a promising therapeutic approach for patients carrying mutations in *SPART*.

## Graphical abstract

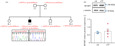

## 1. Introduction

Hereditary spastic paraplegia (HSP) encompasses a large group of rare genetic neurological diseases characterized by degeneration of the upper motor neurons [1,2]. They are all characterized by spasticity and weakness of the lower limbs [2]. The genetic classification of HSP is complex due to multiple inheritance patterns (autosomal dominant, autosomal recessive, X-linked and mitochondrial inheritance) and its heterogeneity, since more than 60 genes are involved in the different forms [3].

Troyer syndrome (SPG20; OMIM no. 275900) is an autosomal recessive form of HSP characterized by additional features such as short stature, cognitive impairment, distal amyotrophy and degeneration of corticospinal tract axons [4–7]. Troyer syndrome is caused by loss-of-function mutations in *SPART*, also known as *SPG20*, which encodes for Spartin [6], a multifunctional protein that consists of a N-terminal microtubule interacting and trafficking (MIT) domain and a C-terminal senescence domain [8]. Through specific modules located at the N-terminal and C-terminal regions, Spartin localizes to microtubules and mitochondria, respectively [8–10]. Moreover, Spartin plays a role in the intracellular trafficking of the epidermal growth factor receptor, in the lipid droplets (LDs) turnover through the recruitment of ubiquitin E3 ligases [11–13], in the bone morphogenetic protein (BMP) signalling and cytokinesis [14]. Renvoise *et al*. have shown that *spg20* knock-out mice developed chondrocyte abnormalities, in the epiphyseal growth plates of bones, suggesting that impairments in cytokinesis might be responsible for the short stature and bone defects observed in Troyer syndrome [14].

Several studies also indicated that Spartin regulates mitochondrial homeostasis [15,16]. Spartin depletion in human neuroblastoma cell lines resulted in a significant decrease in mitochondrial Ca^2+^ uptake and mitochondrial membrane potential [16]. In a yeast model expressing *Drosophila* Spartin, Ring and colleagues demonstrated a cytoprotective function of Spartin connected to glycolytic and respiratory control [15]. We recently reported that homozygous loss-of-function variants of *SPART* caused a severe mitochondrial dysfunction, characterized by Complex I impairment and increased production of mitochondrial reactive oxygen species (ROS) [17].

However, the wide spectrum of molecular defects due to mutant Spartin that result in spastic paraplegia is still partially unknown and Spartin precise role in regulating these cellular processes remains poorly understood.

We performed trio exome sequencing (ES) in a 5-year-old boy presenting with muscle weakness with impaired walking distance, epilepsy, developmental delay and a low normal body height. We identified two compound heterozygous missense variants in *SPART*, inherited from his healthy father and mother, respectively, and found that the mutant fibroblasts derived from the patient showed an altered mitochondrial functionality. We further characterized the mitochondrial impairments in the fibroblasts carrying biallelic missense variants in *SPART* and in a genome-edited neuronal cell model with a homozygous protein truncative mutation. We found that Spartin deficiency affected the mitochondria-associated membrane (MAM) stability, leading to alteration in calcium homeostasis and in the mitochondrial import of nuclear-encoded mitochondrial proteins, with a severe decrease in Coenzyme Q10 (CoQ). Supplementation with CoQ restored ATP/ADP ratio and cell growth rate to control levels, suggesting that CoQ treatment may represent a potential therapeutic approach.

In summary, we were able to further elucidate the cellular role of Spartin and the pathological mechanisms related to loss of Spartin function in Troyer syndrome and to provide a possible therapeutic pathway for the affected individuals.

## 2. Results

### 2.1. Identification of biallelic variants in SPART through ES analysis

**Figure 1.**
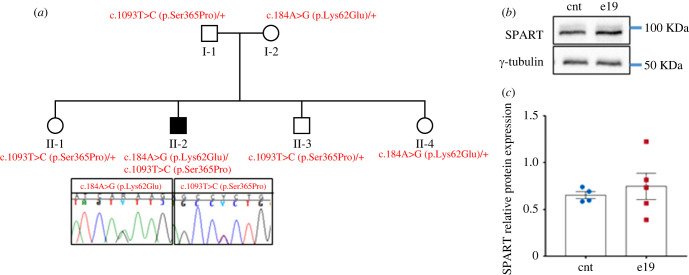
Identification of *SPART* variants. (*a*) Pedigree of the family with mutations in *SPART*. Individuals I-1, I-2 and II-I underwent ES analysis that allowed us to identify two heterozygous variants, c.184A > G, p.(Lys62Glu) and c.1093T > C, p.(Ser365Pro). The variant p.Lys62Glu was inherited from the mother, and the p.Ser365Pro variant from the father. The three healthy siblings were heterozygous for one of the two variants as reported in the pedigree. (*b*) Representative western blot showing Spartin expression in total cell lysates derived from human control (cnt) and patient (e19) fibroblasts. Immunoblotting for Spartin and γ-Tubulin (endogenous control) proteins was performed on the same blot. Cropped images are reported. (*c*) Relative SPART expression normalized on γ-Tubulin (western blot analysis). At least three independent experiments were performed. Unpaired *t*-test with Welch's correction, *p* = ns (means ± s.e.m.).

Trio ES (father–mother-index) was performed for a young boy affected by global developmental delay, muscular hypotonia with exercise intolerance and periventricular white matter changes at brain MRI. Data filtering for autosomal recessive, de novo or X-linked variants identified two compound heterozygous variants in *SPART* (OMIM*607111), inherited from his healthy parents (figure 1*a*). Three healthy siblings were segregated on both variants and while none of those three showed both variants, all were heterozygous for one of the variants (figure 1*a*). The maternally inherited variant c.184A > G, p.Lys62Glu, was present in gnomAD with a very low minor allele frequency (MAF) of 0.00004 and also the second variant c.1093T > C, p.Ser365Pro, inherited from the father, was very rare in gnomAD (MAF = 0.000008).

Both changes were considered variants of uncertain significance (VUS) according to the American College of Medical Genetics and Genomics (ACMG) guidelines, making genetic counselling particularly difficult in the absence of additional functional evidence. However, the patient's symptoms were suggestive of SPG20 syndrome, and we therefore carried out further analyses. We evaluated *in silico* the possible fold changes of the two missense variants with the program AlphaFold [18]. The variant p.Lys62Glu maps in the N-terminal MIT domain and AlphaFold prediction indicated that the Lysine 62, residing in an exposed part of the domain of a MIT-interaction site (electronic supplementary material, figure S1A), would be affected by the charge change due to the Glu62 substitution. Serine 365 was predicted to be an exposed amino acid residue by PSIPRED. DeepREX prediction indicated that it could be a terminal residue of an alpha helix, in a highly flexible region. Therefore, the substitution of the residue with a Proline would structurally alter the alpha helix. Taken together, these data suggested a possible impairment in protein folding. Therefore, we undertook functional studies *in vitro* on primary skin fibroblasts derived from the affected patient (e19), from healthy donors (cnt), and on an already characterized *SPART* cell model, the SH-SY5Y cell line carrying the homozygous variant c.892dupA, in order to evaluate the effects of the biallelic *SPART* variants.

Western blot analysis showed that there were no significant differences in Spartin expression between patient (e19) and control fibroblasts (figure 1*b,c*).

### 2.2. Biallelic missense variants in SPART led to impaired growth and mitochondrial bioenergetics

**Figure 2.**
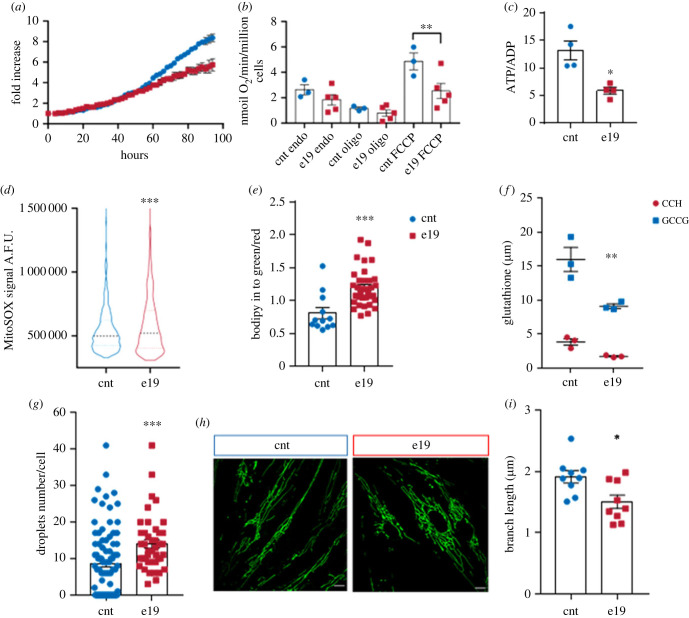
Spartin biallelic variants alter cell proliferation and mitochondrial functionality. (*a*) Cell proliferation analysis assessed via the IncuCyte Live-Cell Assay. Proliferating cells were imaged and analysed every hour over a time range of 96 h. e19 cells (red squares) exhibited a lower cell proliferation rate in comparison with controls (cnt, blue dots). The doubling time of e19 and control fibroblasts was 38.8 h and 28.9 h, respectively. (*b*) Mitochondrial oxygen consumption in endogenous and uncoupled conditions. Respiration was measured in DMEM (basal respiration), in the presence of oligomycin A (non-phosphorylative respiration) and in the presence of FCCP (uncoupled respiration). Compared to controls (cnt, blue dots), e19 fibroblasts (red squares) showed a significantly reduced respiration in the presence of FCCP. At least three independent experiments were conducted; ANOVA for multiple comparisons; **p =0.0029 (means ± s.e.m.). Endo: endogenous basal respiration, oligo: cells treated with oligomycin A. (*c*) ATP/ADP ratio in cellular extracts from control (cnt, blue dots) and e19 cells (red squares). Paired *t*-test, *p = 0.021 (means ± s.e.m., *n* = 4). (*d*) Oxidative stress determination in live cells using MitoSOX. Mutant cells (e19, blue violin plot) showed higher superoxide production versus controls (cnt, red violin plot). At least three independent experiments were performed. Unpaired *t*-test with Welch's correction; ***p = 0.0002. (*e*) Membrane peroxidation determination using BODIPY 581/591 C11. Mutant cells (red squares) showed higher green/red ratio fluorescence intensity signal, indicative of an increased membrane peroxidation status in comparison with controls (blue dots). At least three independent experiments were performed. Unpaired *t*-test with Welch's correction, ***p = 0.0008 (means ± s.e.m.). (*F*) Oxidized and reduced glutathione determination in control (cnt, blue dots) and e19 (red squares) cells. e19 cells showed lower oxidized and reduced glutathione levels versus controls. Three independent experiments were performed. Two-way ANOVA with Sidak's multiple comparison test, GGSH cnt versus e19, **p = 0.0016. (*g*) Lipid droplets determination using Nile red dye. e19 cells (red squares) showed and increased number of lipid droplets per cell in comparison with controls (cnt, blue dots). At least three independent experiments were performed. Unpaired *t*-test with Welch's correction, ***p = 0.0006 (means ± s.e.m.). (*h*) Representative micrographs of control and e19 live fibroblasts stained with MitoTracker Green. (*i*) Morphology analysis performed with ImageJ MiNa plugin. Mutant fibroblasts displayed a perinuclear distribution of mitochondria with significantly shorter branch length in comparison with controls. At least three independent experiments were performed. Unpaired *t*-test with Welch's correction, *p = 0.0138 (means ± s.e.m.).

We assessed the cellular proliferation with the IncuCyte Live-Cell Analysis System over a period of 96 h. Mutant cells exhibited a lower cell proliferation rate in comparison with controls: the doubling time of e19 and control fibroblasts was 38.8 h and 28.9 h, respectively (figure 2*a*). To assess the effect of mutant Spartin on mitochondrial activity, we measured the oxygen consumption rates (OCR) in intact e19 and control cells in basal conditions, after the addition of oligomycin A (to block the ATPase) and of the uncoupler FCCP (to achieve the maximum oxygen consumption rate). e19 cells presented a significantly decreased uncoupled respiration rate in comparison to controls (*p* = 0.0029; figure 2*b*).

We investigated the specific activities of Complex I in isolated mitochondria, and Complex II and Complex II + III activities in cell lysates. The activity of Complex I was significantly decreased by 60% in e19 cells versus controls (*p* = 0.0356; electronic supplementary material, figure S2A). In e19 fibroblasts, the activity of Complex II was significantly decreased by 52%, in comparison to controls (*p* = 0.0397; electronic supplementary material, figure S2B), similarly to what was observed for the cell model carrying the c.892dupA variant (electronic supplementary material, figure S2C). The activities of Complex II + III were also significantly decreased by 65% in e19 cells versus controls (*p* = 0.0385, electronic supplementary material, figure S2D).

In agreement with our data previously obtained in the SH-SY5Y cells homozygous for the c.892dupA variant in SPART [17], e19 cells also showed a significant decrease in ATP/ADP ratio in comparison to controls (*p* = 0.0483; figure 2*c*).

To determine the production of mitochondrial ROS in live cells, we used the fluorogenic probe MitoSox Red, which specifically detects mitochondrial superoxide production [19]. e19 cells showed a significantly higher signal compared to controls (*p* = 0.0002; figure 2*d*). When we stained the cells with the ROS indicator DCFDA, which detects hydroxyl, peroxyl and other ROS, no significant differences were observed between e19 and controls (electronic supplementary material, figure S2E), indicating that mutant Spartin promoted an oxidative stress primarily in the mitochondria. Since lipids are targets of ROS, we stained the cells with the lipid peroxidation sensor BODIPY C11. The oxidation of the dye results in a shift of the fluorescence emission peak from red to green. e19 cells presented a lower red/green ratio in comparison to controls, indicative of increased lipid membrane peroxidation (*p* = 0.0008; figure 2*f*). Considering that mitochondrial ROS production is often associated with mitochondrial hyperpolarization [20–22], we measured the mitochondrial transmembrane potential with the fluorescent TMRM probe and found that in e19 the mitochondrial transmembrane potential was significantly higher than controls (*p* < 0.0001, electronic supplementary material, figure S2I).

We measured the level of reduced (GSH) and oxidized (GSSG) glutathione in e19 and control cell, since GSH is the most significant hydrophilic antioxidant able to remove ROS *via* non-enzymatic reduction, resulting in the oxidized form GSSG. We found that e19 cells presented decreased levels of GSH in comparison to controls (*p* = 0.0016, figure 2*f*; *p* = 0.0426, electronic supplementary material, figure S2J; *p* = 0.0080, electronic supplementary material, figure S2K).

To quantify the presence of LDs in e19 and controls, we labelled the cells with the fluorescent dye Nile Red, finding that mutant fibroblasts had significantly more LDs than controls (*p* = 0.0006; figure 2*g*).

We stained the mitochondria in live e19 and control cells with MitoTracker Green to visualize the network organization. In comparison to control cells (figure 2*h*, left panel), the mitochondrial network in mutant fibroblasts (figure 2*h*, right panel) displayed a perinuclear distribution, with a significantly shorter branch length (*p* = 0.0138; figure 2*i*). The mitochondrial mass, measured using the green mitotracker probe signal, was unchanged in both control fibroblasts versus e19 cells and SY^wt^ versus SY^892dupA^ (electronic supplementary material, figure S2I–L).

**Figure 3.**
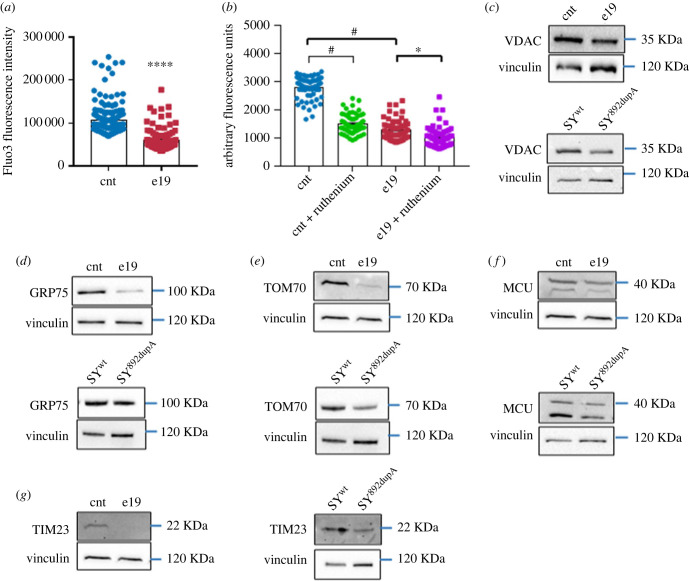
Mutant Spartin alters the mitochondria-associated membrane (MAMs) integrity. (*a*) Intracellular free Ca^2+^ measurement in live control (cnt, blue dots) and mutant (e19, red squares) fibroblasts. Unpaired *t*-test with Welch's correction, *p = 0.001 (means ± s.e.m.). (*b*) Intracellular free Ca^2+^ determination in control (cnt) and mutant (e19) cells in the presence and absence of the mitochondrial Ca^2+^ uniporter inhibitor ruthenium red. Dunn's multiple comparisons test, cnt versus e19 ****p < 0.0001; cnt versus cnt + ruthenium ***p < 0.0001; e19 versus e19 + ruthenium *p = 0.01 (means ± s.e.m.). Cnt, blue dots; cnt + ruthenium, green dots; e19, red squares; e19 + ruthenium, purple squares. (*c–g*) Western blot for VDAC, GRP75, TOM70, MCU and TIM23 in e19 (upper panels) and SY^892dupA^ (lower panels) versus corresponding control cells. Immunoblotting for the different proteins and vinculin (endogenous control) performed on the same blot. Cropped images are reported. The immunoblots are representative of at least three independent experiments.

We measured the intracellular free calcium levels in e19 and control fibroblasts using the Fluo-3 AM probe. As observed previously in SY^c.892dupA^ [17], e19 cells showed a significantly lower fluorescence level than controls (*p* < 0.0001; figure 3*a*). Moreover, to check the involvement of the mitochondrial Ca^2+^ uniporter, we treated the cells with the specific inhibitor of the mitochondrial Ca^2+^ uniporter (MCU), the ruthenium red. In control cells, the fluorescence signal dropped significantly after ruthenium red treatment (cnt versus cnt + ruthenium, *p* < 0.0001; figure 5*b*). In e19 cells treated with ruthenium red, we could measure only a modest decrease in Fluo-3 signal, compared to the untreated controls, suggesting an altered MCU activity (e19 versus e19 + ruthenium, *p* = 0.01; figure 3*b*).

### 2.3. Mutant Spartin affects the mitochondria-associated membrane integrity, leading to an impaired mitochondrial protein import and decreased CoQ biosynthesis

Considering that in mutant cells, we observed an alteration in Ca^2+^ levels and that Spartin localizes on the OMM and interacts with GRP75, we hypothesized a role for Spartin in the formation/stabilization of the complex IP_3_R-GRP75-VDAC-MCU. This is a key group of proteins in mitochondrial-associated membranes (MAMs) regulating the transport of Ca^2+^ from ER to mitochondria [23]. To investigate this mechanism, we assessed whether alterations in the expression of the complex subunits were present in all mutant versus control cells: actually, western blot analyses revealed a reduced expression of VDAC, GRP75, TOM70 and MCU in e19 and SY^c.892dupA^ compared to their corresponding control cells (*p* = 0.0337 for e19, *p* = 0.030 for SY^c.892dupA^, figure 3*c*; *p* = 0.0089 for e19, *p* = 0.0020 for SY^c.892dupA^, figure 3*d*; *p* = 0.0467 for e19, *p* = 0.0149 for SY^c.892dupA^, figure 3*e*; *p* = 0.0010 for e19, *p* = 0.0367 for SY^c.892dupA^, figure 3*f*; electronic supplementary material, figure S3A–D).

Compared to the corresponding controls, all mutant cells showed a strong reduction in TIM23, a TOM70-associated subunit in the TIM-TOM complex regulating the import of nuclear-encoded mitochondrial proteins (*p* = 0.0006 for e19 cells and *p* = 0.0179 for SY^c.892dupA^, figure 3*g*; electronic supplementary material, figure S3E).

**Figure 4.**
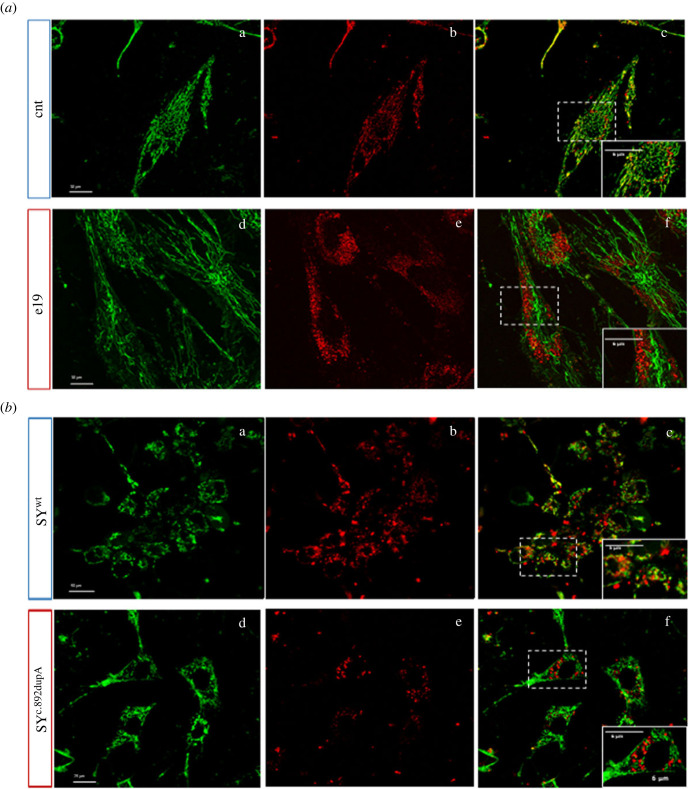
Mutant Spartin impairs mitochondrial protein import. (*a*) Control and e19 fibroblasts stained with MitoTracker Green (a and d) and an RFP that targets the mitochondria (CellLight Mitochondria-RFP, b and e). Panels c and f represent merged images. Insets in c and f are magnified images of the region outlined with dashed line, with scale bar representing 6 µm; scale bars in a and d represent 30 µm*.* (*b*) SY^wt^ and SY^892dupA^ stained with MitoTracker Green (a and d) and an RFP that targets the mitochondria (CellLight Mitochondria-RFP, b and e). Panels c and f represent merged images. Insets in c and f are magnified images of the region outlined with dashed line, with scale bars representing 5 and 6 µm, respectively; scale bar in a represents 50 µm, and scale bar in d represents 25 µm. Colocalization of green and red signals was observed in the mitochondria of control cells, whereas the signals did not colocalize in mutant cells e19 and SY^892dupA^ (lack of import of mitochondrial RFP).

Considering the observed reduction in mutant cells of TOM70 and TIM23, both involved in the mitochondrial import machinery, and the decrease in Complex II activity, which is completely nuclear-encoded, both in e19 cells (electronic supplementary material, figure S2B) and in SY^c.892dupA^ (*p* = 0.0155, electronic supplementary material, figure S2C), we investigated whether mutations in Spartin could cause an alteration in the import of nuclear-encoded mitochondrial proteins. We co-stained control and mutant cells with MitoTracker Green and Mitochondria-RFP probes (figure 4*a,b*). In control cells (control fibroblasts and SY^wt^), the green signal of MitoTracker Green co-localized with the red signal of mitochondrial RFP (figure 4*a*, panels c,d*;* figure 4*b*, panels c,d), whereas in e19 and SY^c.892dupA^ cells the red and green signals did not co-localize (figure 4*a*, panels g,h*;* figure 4*b*, panels g,h), suggesting that RFP failed to be imported in mitochondria.

**Figure 5.**
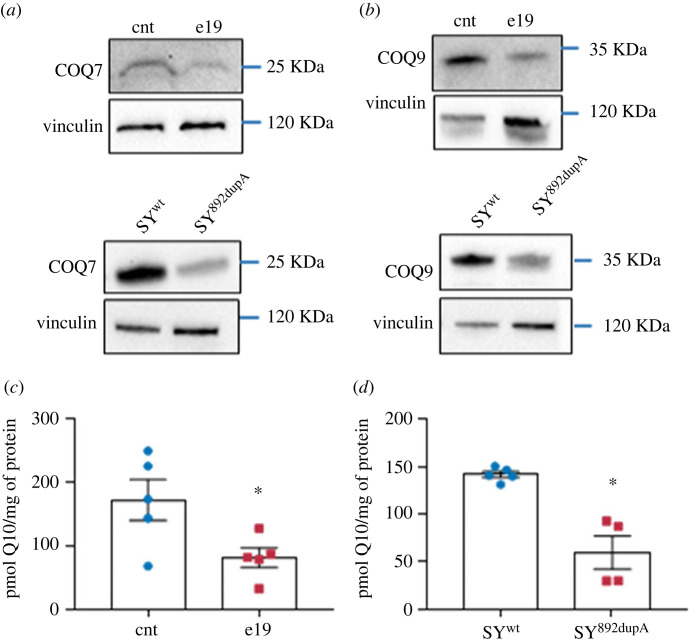
Mutant Spartin impairs CoQ biosynthesis. (*a*) Western blot analysis for COQ7 and COQ9 proteins in e19 (upper panels) and SY^892dupA^ (lower panels) cells versus control (cnt) cell lysates. Immunoblotting for COQ7 and vinculin (endogenous control) was performed on the same blot. Cropped images are reported. The immunoblots are representative of three independent experiments. (*c*) Total cellular CoQ in control and e19 fibroblasts. At least three independent experiments conducted. Unpaired *t*-test with Welch's correction, *p = 0.045 (means ± s.e.m.). (*d*) Total cellular CoQ in SY^wt^ and SY^892dupA^ cells. At least three independent experiments conducted. Unpaired *t*-test with Welch's correction, *p = 0.0165 (means ± s.e.m.).

The mitochondrial import is relevant for many mitochondrial protein complexes, such as the ‘complex Q’, which performs the final stage of CoQ biosynthesis and comprises different enzymes, including COQ7 and COQ9. In all mutant cells (e19 and SY^c.892dupA^), we found a reduced expression of both COQ7 and COQ9 (*p* = 0.0250 for e19, *p* = 0.0127 for SY^c.892dupA^, figure 5*a*; and *p* = 0.0447 for e19, *p* = 0.0143 for SY^c.892dupA^, figure 5*b*; electronic supplementary material, figure S3F,G, respectively). Accordingly, we found a significant reduction in CoQ content both in e19 and SY^892dupA^ mutant cells compared to their corresponding control cells (*p* = 0.0455 for e19, figure 5*c*; *p* = 0.0165 for SY^c.892dupA^, figure 5*d*, respectively).

### 2.4. Wild-type SPART transfection recovers the bioenergetics deficiency in mutant e19 cells

**Figure 6.**
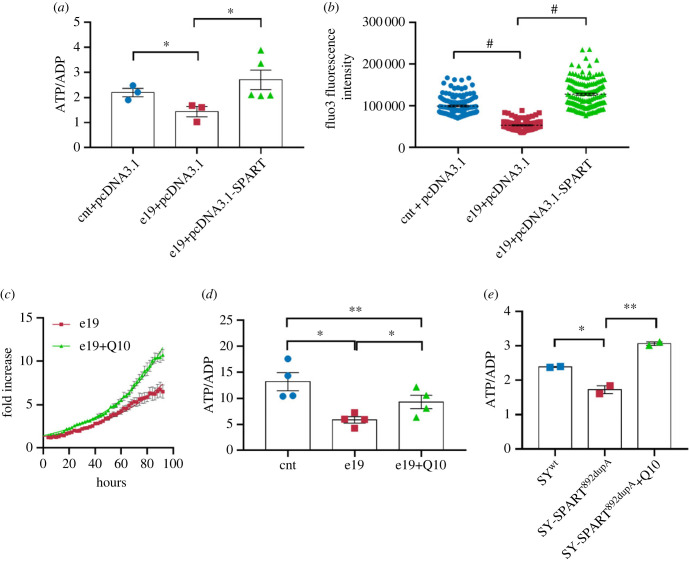
Rescue of CoQ deficiency and bioenergetic impairments in mutant cells by wild-type *SPART* transfection or CoQ supplementation. (*a*) ATP/ADP ratio in control (cnt-pcDNA3.1, blue dots), e19 mutant (e19-pcDNA3.1, red squares) and e19 mutant cells re-expressing Spartin (e19-pcDNA3.1-SPART, green triangles). At least three independent experiments were conducted. Unpaired *t*-test with Welch's correction; cnt + pcDNA3.1 versus e19 + pcDNA3.1 *p = 0.0483 (means ± s.e.m.), e19 + pcDNA3.1 versus e19 + pcDNA3.1-SPART *p = 0.0288 (means ± s.e.m.). (*b*) Intracellular free Ca^2+^ in live control (cnt-pcDNA3.1, blue dots), mutant (e19-pcDNA3.1, red squares) and mutant cells re-expressing Spartin (e19-pcDNA3.1-SPART, green triangles). At least three independent experiments conducted; Games–Howell's multiple comparisons test, cnt-pcDNA3.1 versus e19-pcDNA3.1, ****p < 0.0001; e19-pcDNA3.1 versus e19-pcDNA3.1-SPART, ****p < 0.0001, (means ± s.e.m.). (*c*) Cell proliferation analysis in mutant cells (e19) incubated with 100nM CoQ, imaged and analysed every hour for 96 h. Unpaired *t*-test with Welch's correction, *p = 0.05 (means ± s.e.m.). (*d*) ATP/ADP ratio determination in control (cnt, blue dots), e19 (red squares), and mutant cells supplemented with CoQ (green triangles). At least three independent experiments conducted. Paired *t*-test, *p = 0.03 (means ± s.e.m.). (*e*) ATP/ADP ratio in control SH-SY5Y cells (SY^wt^, blue dots), SH-SY5Y cells with c.892dupA homozygous change (SY-SPART^892dupA^, red squares) and SY-SPART^892dupA^ + Q10 (green triangles). Tukey's multiple comparisons test, SY-SPART^892dupA^ versus SY_SPART^892dupA^ +Q10, **p = 0.0021 (means ± s.e.m.).

To prove that the observed defects in e19 fibroblasts were specifically caused by the biallelic missense variants of Spartin, we transiently transfected wild-type Spartin in e19 cells (electronic supplementary material, figure S4A). e19 cells transfected with wild-type Spartin showed an ATP/ADP ratio similar to the controls, in comparison with e19 cells transfected with the empty vector (*p* = 0.0288; figure 6*a*). Moreover, e19 cells transfected with wild-type Spartin showed a significant recovery of intracellular free Ca^2+^, in comparison with e19 cells transfected with the empty vector (e19-pcDNA3.1 versus e19-pcDNA3.1-SPART, *p* < 0.0001; figure 6*b*), with intracellular free Ca^2+^ levels similar to those measured in control cells (figure 6*b*). These data perfectly matched the ones observed in the SPART^c.892dupA^ cells transfected with wild-type SPART, supporting a causative role of the missense changes in inducing the observed deficits [17].

### 2.5. Coq supplementation rescue the bioenergetics deficiency in mutant cells

Based on the observed CoQ deficiency in all mutant cells, we supplemented control and mutant cells with CoQ and evaluated its effects in term of growth rate, oxygen consumption and ATP production.

CoQ treatment improved e19 doubling time, from 36.82 to 30.33 h (figure 6*c*), whereas it did not affect control cells (electronic supplementary material, figure S4B). The integrated activity of Complexes II and III, mediated by the electron transfer capacity of CoQ, was stimulated in e19 by CoQ supplementation (controls versus e19 + Q10 *p* = ns; electronic supplementary material, figure S4C). CoQ supplementation restored the ATP/ADP ratio in e19 cells (e19 versus e19 + Q10, *p* = 0.03; e19 + Q10 versus cnt, *p* = ns; figure 6*d*), and in the genome-edited SH-SY5Y^c.892dupA^ cells to control levels (SY^c.892dupA^ versus SY^892dupA^ + Q10, *p* = 0.0021; figure 6*e*). Moreover, CoQ-treated e19 and SY^892dupA^ mutant cells displayed a higher FCCP-induced respiration in comparison with the corresponding untreated mutant cells, indicative of an improved maximal respiratory capacity for ATP production.

## 3. Discussion

The increased availability of NGS holds the potential of higher diagnostic yield, but it can also increase the likelihood of discovering uncertain results. The ACMG advocated for the use of a five-tier classification system for variant interpretations: benign, likely benign, variant of uncertain significance (VUS), likely pathogenic and pathogenic [24]. VUSs are genetic variants lacking sufficient evidence to indicate whether they are benign or pathogenic, and thus are not intended to inform clinical decision-making [25]. Establishing a firm diagnosis is particularly critical in the context of prenatal testing or management of severely affected pediatric patients. In a routine diagnostic setting, this task is especially challenging in affected individuals in whom broad unbiased sequencing approaches such as ES or genome sequencing usually result in a number of rare VUS missense changes [26–28]. Nevertheless, as more research is conducted on these variants, they might be upgraded to pathogenic or downgraded to benign, thereby emphasizing the importance of functional studies in the diagnostic variant reclassification process [29,30]. In this framework, we present the identification of two rare missense variants in *SPART* in a young male proband with clinical features consistent with a diagnosis of Troyer syndrome including low normal stature, muscle weakness, impaired walking distance, developmental delay and MRI features compatible with the findings reported previously for this disorder [5,31], and epilepsy as likely an extension of the phenotype, since no other causative variants in epilepsy-related genes were identified via ES. Carrier testing and segregation analyses were compatible with an autosomal recessive model of inheritance and allele frequencies of the changes in an in-house database and public repositories did not exclude a putative disease-causal role at the time of the study. To understand the functional relevance and cellular consequences of the *SPART* variants, we performed extensive biochemical and bioenergetics studies on patient-derived primary fibroblasts and *SPART* mutant cell models. We observed a different mitochondrial network organization and a severe bioenergetic impairment, in terms of decreased OXPHOS activities and ATP production, in concordance with our previous studies for the loss-of-function *SPART* c.892dupA variant [17].

Actually, mutant e19 cells were characterized by decreased ATP-linked respiration, decreased respiratory spare capacity and higher membrane potential (mt*ΔΨ*) versus control cells. As observed in other mitochondrial disorders, the mitochondrial hyperpolarization promoted superoxide production and oxidative stress in e19 cells [32,33]. The observed cellular phenotypes were rescued by expression of wild-type *SPART*.

Together, these findings provide strong arguments for a functional relevance of the identified *SPART* missense variants and support the hypothesis of SPART deficiency being the molecular defect underlying the patient's disease presentation.

In our previous study, we have shown the role of Spartin in mitochondrial homeostasis and Ca^2+^ levels [17]. Herein, we undertook an in-depth analysis on the effect of mutant Spartin on the mitochondrial import of nuclear-encoded proteins and on the structure of the MAMs, a physical association between the endoplasmic reticulum (ER) and mitochondria [34]. MAMs, allowing the bidirectional crosstalk between mitochondria and ER, participate in fundamental biological processes, including lipid and calcium homeostasis, mitochondrial dynamics, autophagy, mitophagy, ER stress, inflammation and apoptosis [23,34–38]. The connection between ER and mitochondria is formed by an ER subdomain, the outer mitochondrial membrane (OMM) and more than 68 proteins [39], including the ones mediating Ca^2+^ transport from the ER to the mitochondria. Strikingly, in mutant cells, we observed a significant reduction of VDAC, GRP75, MCU, TOM70 and TIM23, part of the TIM complex, and an altered Ca^2+^ level specifically rescued by wild-type *SPART* re-expression. It is noteworthy that the mutant cells are insensitive to the MCU complex inhibitor ruthenium red, further indicating a role of SPART on calcium homeostasis.

**Figure 7.**
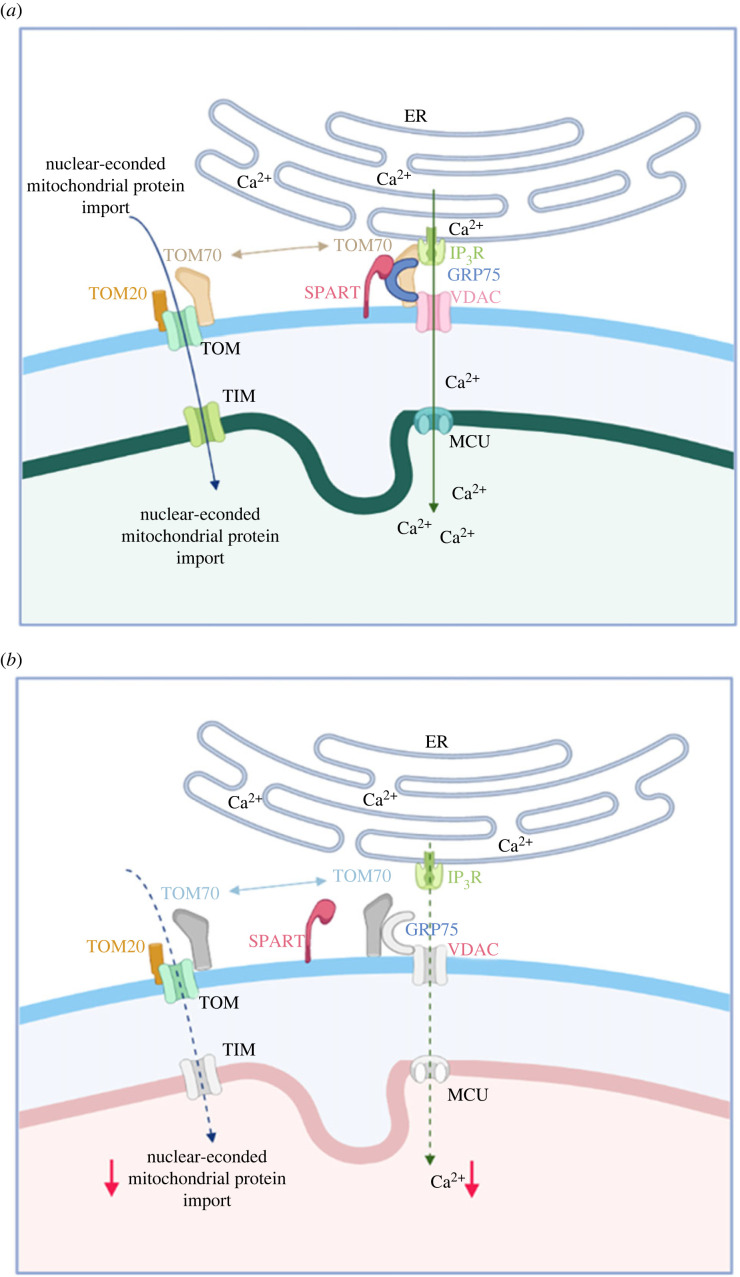
Model for the molecular role of wild-type and mutant Spartin at the MAMs. (*a*) *Wild-type Spartin*. The protein core complex mediating the Ca^2+^ transport from the ER to the mitochondria comprehends IP_3_R (green), located in the ER, VDAC (light pink), located in the OMM and mediating the uptake of Ca^2+^ into mitochondria, GRP75 (blue), which forms a bridge between IP_3_R and VDAC, and the mitochondrial calcium uniporter (MCU, turquoise), the calcium channel localized in the inner mitochondrial membrane. Spartin (red) localizes to the OMM and binds to GRP75. TOM70 shifts between the nuclear-encoded mitochondrial protein import complex and the ER-mitochondria contacts, recruiting IP_3_R, and promoting inter-organelle Ca^2+^ transfer, bioenergetics, and cell proliferation. (*b*) *Absent Spartin*. When Spartin is mutated/absent, there is a reduced expression of VDAC, GRP75, MCU, TOM70 and TIM23, part of the TIM complex and an altered Ca^2+^ transport leading to defects in the import of nuclear-encoded mitochondrial proteins, with an impaired metabolism and altered mitochondrial functions as final effects. Figures created with Biorender.com.

GRP75 is one of the few known interactors of SPART, which is located to the OMM associated with cardiolipins [16]. On the mitochondrial membranes, the combined action of the mitochondrial translocase of the outer membrane (TOM) and the translocase of the inner membrane (TIM) coordinates the post-translational import of nuclear-encoded mitochondrial proteins (figure 7*a*) [40,41]. TOM70 shifts between the nuclear-encoded mitochondrial protein import complex and the ER-mitochondria contacts, recruiting IP_3_R, and promoting inter-organelle Ca^2+^ transfer, bioenergetics and cell proliferation [41].

The decrease in the nuclear-encoded mitochondrial proteins of the MAMs suggests that mutant *SPART* alters the mitochondrial import of nuclear-encoded proteins (figure 7*b*). Indeed, when we transfected mutant and control cells with a mitochondrial-labelled red fluorescent protein, followed by staining with the MitoTracker Green dye, we observed in the mutant cells separated green and red fluorescent signals, while the control cells exhibited an overlapping signal. This suggests that mutant cells fail to import the red fluorescent protein in the mitochondria. To our knowledge, this is the first report of an impaired mitochondrial transport attributable to variants in *SPART*. The mitochondrial transport of nuclear-encoded proteins is crucial for the assembly and function of many mitochondrial multiprotein complexes, including OXPHOS complexes and the enzymes forming the ‘Complex Q’, which are involved in the final stage of Coenzyme Q (CoQ) biosynthesis [42]. We found a significant reduction in the expression of the ‘complex Q' enzymes COQ7 and COQ9 in the mutant versus control cells [43] and, consequently, a reduced CoQ content. The decrease in CoQ can profoundly affect the bioenergetics and redox homeostasis of *SPART*-mutant cells, as CoQ is an obligate electron carrier in the electron transfer complex (ETC) and a lipophilic antioxidant [44]. Interestingly, we were able to rescue *in vitro* the growth rate and ATP levels of mutant cells by exogenous CoQ supplementation. Recovery of ATP levels after CoQ supplementation was comparable to that observed in mutant cells transfected with wild-type Spartin. The primary cause of mitochondrial dysfunction due to SPART mutations could be either due to OXPHOS defects or increased ROS production, i.e. whether an impaired protein import led to the loss of respiratory complex assembly or lack of ubiquinone-producing enzymes. Nevertheless, since we could prove that CoQ supplementation rescued the cell bioenergetics, the latter mechanism seems the most likely.

In addition, CoQ treatment might also improve cell growth by relieving the severe mitochondrial oxidative stress present in the mutant cells, as indicated by the high mitochondrial superoxide production and reduced glutathione levels [42].

It has been observed that the administration of CoQ has no absolute contraindication and very rare adverse effects, with less than 1% of patients facing mild dose-related gastrointestinal discomfort [45]. Intriguingly, CoQ administration has been used for treating neurological and neurodegenerative disorders with defective mitochondrial function and for mitochondrial disorders, which are often multisystemic, with a prevalent impairment of high-energy demanding cells, such as neurons and muscle cells [46,47]. Our data suggest that patients with Troyer syndrome might benefit of the same therapy, the mitochondrial impairment being a prominent cellular feature in this disease.

In conclusion, through NGS coupled to molecular phenotyping, we were able to assign a functional relevance and diagnostic potential to variants otherwise classified of unknown significance. Moreover, the identification of ‘druggable' altered pathways such as the CoQ biosynthesis paves the way to an *in vivo* treatment for a rare but severe disease such as Troyer syndrome.

## 4. Material and methods

### 4.1. Subject

A 5-year-old male patient was referred due to global developmental delay and muscular hypotonia with a predominant proximal muscular weakness. Additionally, he suffered from leg pain after walking. He was born spontaneously after a reportedly uneventful pregnancy and his postnatal period was uneventful. One younger and two older siblings are reportedly healthy. The patient was able to sit and walk by the age of 2 years. He started talking in single words at the age of 2.5 years; his speech remained far behind normal development, and at the age of 6 he was able to build simple sentences. He showed a bilateral *pes planovalgus*, hypotrophic calfs and brisk reflexes in his lower extremities. He also presented a mild conductive hearing loss but did not require hearing aids. He was 109 cm (4th percentile) in height, weighed 17 kg (4th percentile) and had a head circumference of 51 cm (20th percentile). Metabolic screening in blood and urine showed normal results. A Snijders–Oomen non-verbal intelligence test (SON-IQ) at the age of 5 years and 8 months showed an IQ of 50. Additionally, he suffered from epilepsy with absences, for which he was treated with levetiracetam, and hyperopia. He showed no facial dysmorphisms.

### 4.2. Next generation sequencing analysis

ES was performed on genomic DNA of the index patient and his healthy parents as described previously [48]. In brief, coding genomic regions were enriched using the SureSelectXT Human All Exon Kit V7 (Agilent Technologies, Santa Clara, CA, USA) and subsequently sequenced as 2 × 100 bp paired-end reads on a NovaSeq6000 system (Illumina, San Diego, CA, USA). Variant calling was performed using the megSAP pipeline (https://github.com/imgag/megSAP) with variant prioritization including filtering for rare (MAF less than 1% in gnomAD) autosomal recessive, de novo or X-linked variants. Carrier testing of additional family members was conducted by Sanger sequencing.

### 4.3. Cell line cultures

Control and patient's dermal fibroblasts derived from skin biopsies and the SH-SY5Y cell models (wild-type and genome-edited to insert the *SPART* c.892dupA [17]) were cultured in Dulbecco's modified Eagle's medium (DMEM, Euroclone, Milan, Italy) supplemented with 10% (v/v) fetal bovine serum, 100 U ml^−1^ penicillin and 100 mg ml^−1^ streptomycin (Millipore Sigma, Burlington, MA, USA). Cells were grown in a humidified incubator with 95% air and 5% CO_2_ at 37°C. Where indicated, cells were supplemented with 100 nM of a phytosome formulation of CoQ (Q10, Indena S.R.L, Milan, Italy) [49–52].

## 5. Methods

Detailed descriptions of western blotting, cell proliferation, mitochondrial bioenergetics, NAD(P)H measurements, ROS, glutathione analysis, lipid peroxidation and quantification of LDs, Ca^2+^ transport, mitochondrial protein import and CoQ measurement are reported in the electronic supplementary material and methods section.

### 5.1. Statistical analysis

ANOVA and Student's *t*-tests and appropriate corrections were performed as indicated in the corresponding Results sections and figure legends, using GraphPad Prism software v.8.0 (GraphPad). *p*-values ≤ 0.05 were considered statistically significant.

## Data Availability

Data have been reported in the Results section and methods are presented either in the main text or in the electronic supplementary material [53] and methods section.
